# Characterization of glucose‐related metabolic pathways in differentiated rat oligodendrocyte lineage cells

**DOI:** 10.1002/glia.22900

**Published:** 2015-09-09

**Authors:** Ana I. Amaral, Mussie G. Hadera, Joana M. Tavares, Mark R. N. Kotter, Ursula Sonnewald

**Affiliations:** ^1^ Anne McLaren LaboratoryWellcome Trust‐Medical Research Council Cambridge Stem Cell Institute and Department of Clinical Neurosciences, University of CambridgeCambridgeCB2 0SZUnited Kingdom; ^2^ Department of Neuroscience, Faculty of MedicineNorwegian University of Science and TechnologyTrondheim7491Norway; ^3^ Department of Drug Design and Pharmacology, Faculty of Health and Medical SciencesUniversity of CopenhagenCopenhagen2100Denmark

**Keywords:** oligodendroglia, energy metabolism, glucose, ^13^C, mitochondria, glycolysis, acetate, pyruvate carboxylation

## Abstract

Although oligodendrocytes constitute a significant proportion of cells in the central nervous system (CNS), little is known about their intermediary metabolism. We have, therefore, characterized metabolic functions of primary oligodendrocyte precursor cell cultures at late stages of differentiation using isotope‐labelled metabolites. We report that differentiated oligodendrocyte lineage cells avidly metabolize glucose in the cytosol and pyruvate derived from glucose in the mitochondria. The labelling patterns of metabolites obtained after incubation with [1,2‐^13^C]glucose demonstrated that the pentose phosphate pathway (PPP) is highly active in oligodendrocytes (approximately 10% of glucose is metabolized via the PPP as indicated by labelling patterns in phosphoenolpyruvate). Mass spectrometry and magnetic resonance spectroscopy analyses of metabolites after incubation of cells with [1‐^13^C]lactate or [1,2‐^13^C]glucose, respectively, demonstrated that anaplerotic pyruvate carboxylation, which was thought to be exclusive to astrocytes, is also active in oligodendrocytes. Using [1,2‐^13^C]acetate, we show that oligodendrocytes convert acetate into acetyl CoA which is metabolized in the tricarboxylic acid cycle. Analysis of labelling patterns of alanine after incubation of cells with [1,2‐^13^C]acetate and [1,2‐^13^C]glucose showed catabolic oxidation of malate or oxaloacetate. In conclusion, we report that oligodendrocyte lineage cells at late differentiation stages are metabolically highly active cells that are likely to contribute considerably to the metabolic activity of the CNS. GLIA 2016;64:21–34

## Introduction

Oligodendrocytes make up a large proportion of the cells in the central nervous system (CNS). Although oligodendrocytes are vulnerable to low energy conditions (Lyons and Kettenmann, 1998; Yan and Rivkees, 2006), their metabolic properties, including their glucose metabolism, have not been investigated in depth (Amaral et al., 2013). In contrast, the metabolic interactions between neurons and astrocytes, have received considerable attention since their discovery in the 1970s (van den Berg and Garfinkel, 1971). Specifically, the shuttling of glutamine–glutamate–γ‐Aminobutyric acid (GABA) between astrocytes and neurons is thought to be fundamentally important for neuronal function. Because neurons themselves are unable to generate essential precursors of glutamate, GABA, and aspartate, they depend on the supply of glutamine as a precursor from astrocytes for the production of neurotransmitters (glutamate in 90% of the synapses, and GABA in 5%) (Attwell and Laughlin, 2001). In this context, glucose plays a central role as the key molecular building block that is used to synthesize glutamate, GABA, and aspartate.

Glucose is primarily metabolized to pyruvate via glycolysis in the cytosol. Stepwise conversion of a single glucose molecule into two pyruvate molecules generates two molecules of Adenosine triphosphate (ATP) . These reactions are not oxygen dependent. Glucose metabolism can also take an alternative route via a biosynthetic pathway termed *pentose phosphate pathway* (PPP). This complex detour bypasses several steps of glycolysis. In the first, *oxidative phase* of the PPP, NADP+ is converted into Nicotinamide adenine dinucleotide phosphate (NADPH). NADPH acts as a reducing agent that may participate in lipid and steroid synthesis or in the regeneration of glutathione and thioredoxin, which are involved in the cell's defense mechanism against oxidative stress. In the second phase of the PPP, 5‐carbon sugars are nonoxidatively synthetized. The PPP joins the glycolytic pathway at the level of glyceraldehyde‐3‐phosphate (GA3P) and fructose‐6‐phosphate (fructose‐6P). Fructose‐6P is subsequently converted into pyruvate, which constitutes the endpoint of both glycolysis and the PPP.

In the presence of oxygen, the pyruvate produced by glycolysis or by the PPP can be converted to acetyl CoA by the pyruvate dehydrogenase (PDH) complex, and subsequently metabolized in the mitochondrial tricarboxylic acid (TCA) cycle, to further produce ATP via coupling to the mitochondrial electron transport chain. Alternatively, pyruvate can be (reversibly) converted into lactate in the cytosol, which results in the production of NAD+ from NADH. Net synthesis of TCA cycle intermediates and related compounds, including glutamate and glutamine, depend on *anaplerotic* replenishment of intermediates in the TCA cycle. In the brain, this is mediated by pyruvate carboxylase (PC; Patel, 1974). Pyruvate carboxylation was shown to be absent in neurons, but present in astrocytes (Cesar and Hamprecht, 1995; Hertz et al., 1980; Shank et al., 1985; for review, see Sonnewald and Rae, 2010). Consequently, neurons are thought to depend on astrocytes as an external source of glutamine for the production of neurotransmitters. Conversion of pyruvate by PC generates a “new” molecule of oxaloacetate. Oxaloacetate may subsequently condense with acetyl CoA to synthesize the TCA cycle intermediate citrate, which, after several steps, is converted to α‐ketoglutarate, from which glutamate can be formed by transamination or deamination. In a subsequent step, glutamine synthetase, which is known to be expressed in astrocytes (Martinez‐Hernandez et al., 1977; Norenberg and Martinez‐Hernandez, 1979), is able to convert glutamate into glutamine (see Fig. 1 in Amaral et al., 2013).

**Figure 1:**
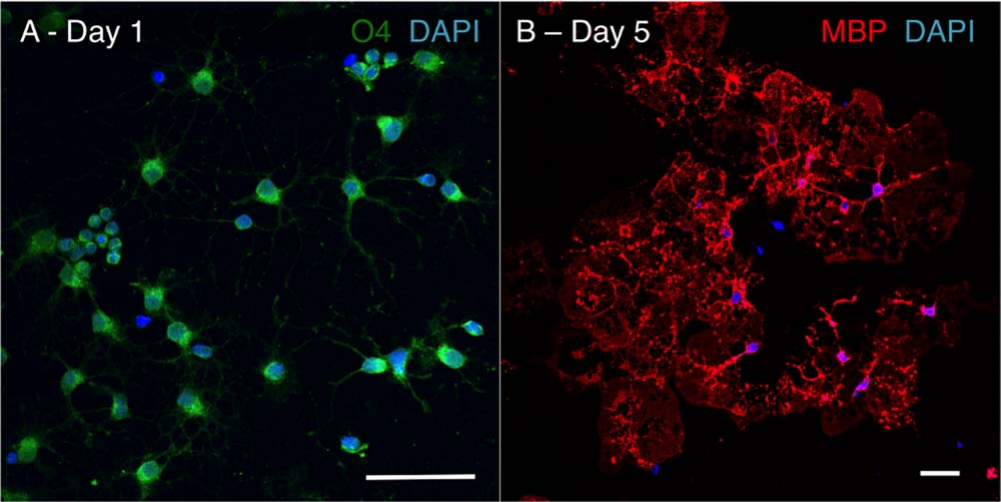
Purity of the primary cultures of rat oligodendrocytes. Oligodendrocyte precursor cells were isolated from mixed glia cultures and cultured in Sato's medium + 0.05% FCS to induce differentiation. At day 1 of differentiation, more than 93% of the cells expressed the oligodendroglial lineage marker O4 (**A**) and at 5 days of differentiation, approximately 65% of the cells expressed myelin basic protein (MBP), a marker of mature oligodendrocytes (**B**). Scale bars, 50 µm. [Color figure can be viewed in the online issue, which is available at wileyonlinelibrary.com.]

In the gray matter, glutamate, released from neuronal synapses during glutamatergic neurotransmission, is mainly taken up by astrocytes (Gegelashvili and Schousboe, 1997, 1998). The drain of glutamate from signalling neurons is subsequently compensated by a reverse flow of glutamine from astrocytes back to the neurons. This cross flow of glutamate and glutamine is often referred to as the glutamate—glutamine cycle (McKenna et al., 2012; see Fig. 1 in Amaral et al., 2013). Because glutamine released by astrocytes also functions as a precursor for the production of the inhibitory neurotransmitter GABA via conversion to glutamate (Reubi et al., 1978; Sonnewald et al., 1993b), metabolic interactions between astrocytes and neurons are thought to consist of a glutamate–glutamine and a glutamine–glutamate–GABA cycle.

How can oligodendrocytes contribute to the metabolic interactions in the CNS? We have argued that, instead of being restricted to closed‐loop interactions between astrocytes and neurons, intercellular shuttling of metabolites may occur between all three major cell groups of the CNS: neurons, astrocytes, and oligodendrocytes (Amaral et al., 2013). The limited understanding of the metabolic role of oligodendrocytes in the brain was further highlighted in two recent studies, which, for the first time, proposed a link between glycolytic metabolism in oligodendrocytes and axonal integrity and function (Funfschilling et al., 2012; Lee et al., 2012).

The aim of this study was to elucidate basic metabolic pathways for glucose catabolism and the anaplerotic replenishment of TCA cycle intermediates in oligodendrocytes. For this purpose, we incubated primary cultures enriched for mature oligodendrocytes in medium containing [1,2‐^13^C]glucose, [1,6‐^13^C]glucose, [1‐^13^C]lactate, or [1,2‐^13^C]acetate and analyzed cell extracts and medium using mass spectrometry or nuclear magnetic resonance (NMR) spectroscopy. We found that oligodendrocytes have extensive PPP activity. Furthermore, oligodendrocytes were able to anaplerotically replenish the TCA cycle, via pyruvate carboxylation, and cataplerotically recycle pyruvate. Our data also show that oligodendrocytes are able to convert [1,2‐^13^C]acetate into acetyl CoA. Our results establish hallmarks of the cellular metabolism of oligodendrocytes under physiological conditions. They may also be useful for future studies investigating altered oligodendrocyte function and injury in diseases that involve glutamate toxicity and impaired mitochondria function, including, for example, hypoxic–ischemic damage, and multiple sclerosis (MS; Kostic et al., 2013; Mifsud et al., 2014; Pitt et al., 2000; Simonishvili et al., 2013).

## Materials and Methods

### Materials

Cell culture reagents were purchased from Sigma (Dorset, UK)—Dulbecco's modified eagle's medium (DMEM), minimum essential medium eagle (MEM), l‐glutamine, poly‐l‐lysine (PLL), papain—or Life Technologies (Paisley, UK)—fetal bovine serum (FBS), penicillin‐streptomycin (pen‐strep), trypsin‐Ethylenediaminetetraacetic acid (EDTA), phosphate buffered saline (PBS). ^13^C‐labelled compounds were obtained from Cambridge Isotope Laboratories, MA. The mass spectrometry derivatization reagents MTBSTFA (*N*‐methyl‐*N*‐(*tert*‐butyldimethylsilyl) trifluoroacetamide), MSTFA (*N*‐Methyl‐*N*‐(trimethylsilyl) trifluoroacetamide) and the t‐BDMS‐Cl (*tert*‐butyldimethylchlorosilane) were purchased from Regis Technologies (Morton Grove, IL). All other chemicals were of the purest grade available from Sigma (Dorset, UK).

### Preparation of Primary Oligodendrocyte Precursor Cell Cultures

Primary mixed glia cultures were isolated from neonatal Sprague Dawley rat (postnatal day 0–2) forebrains following a standard protocol (Baer et al., 2009). Pups were euthanized according to “Schedule 1” regulations from the Home Office Animal Procedures Committee UK. Cells were cultured for 10–15 days in DMEM supplemented with 10% FBS, 1% pen‐strep, and 4 mM glutamine, and kept under a humidified atmosphere at 37°C and 7% CO_2_. Oligodendrocyte precursor cells (OPCs) were subsequently isolated using a step‐based shake‐off protocol and cultured in Sato's medium on PLL‐coated plates (Baer et al., 2009). To induce differentiation, OPCs were cultured in Sato's medium supplemented with 0.5% fetal calf serum (FCS). The cell culture medium was replaced by fresh medium at day two of differentiation. For all *in vitro* experiments, only cultures with >93% purity (determined based on O4 immunostaining) were used (Fig. 1). Quantification of the number of glial fibrillary acidic protein (GFAP)‐positive cells after 5 days of differentiation, indicated that astrocytes comprised 7–10% of total cells (data not shown).

### Preparation of Rat Cortical Astrocyte Cultures

Astrocytes were prepared from the same mixed glia cultures used for OPC isolation, following an adapted protocol described in Amaral et al. (2014). After the shake‐off (which eliminates microglia and most oligodendrocytes from the cultures), the remaining cells, highly rich in proliferative astrocytes, were seeded into new T75 flasks (1:3 dilution) using DMEM supplemented with 10% (v/v) FBS, 4 mM glutamine, and 1% (v/v) pen‐strep and allowed to reach confluence (approx. 5–7 days). Then, cells were collected with trypsin‐EDTA and seeded in 6 well plates at a density of 5 × 10^4^ cells/well for the [1‐^13^C]lactate experiments. Experiments were performed when cells reached confluence.

### Incubations with ^13^C‐Labelled Compounds

Mature OPC cultures (after 5 days in differentiation medium) were cultured in 6 well plates at a cell density of 4 × 10^5^ cells/well. Prior to incubation, cells were washed once with PBS and incubated with 2 mL Sato's medium prepared from a glucose, glutamine, and pyruvate‐free DMEM (Sigma D5030) supplemented with 0.5% FCS and 2 mM [1,6‐^13^C]glucose, 2 mM [1,2‐^13^C] glucose + 1 mM glutamine, 2 mM [1,2‐^13^C]acetate + 1 mM glutamine, or 5 mM [1‐^13^C]lactate + 2 mM glucose + 1 mM glutamine for 24 h. Astrocytes were washed once with PBS and incubated with 2 mL DMEM (Sigma D5030) supplemented with 4 mM [1‐^13^C]lactate + 2 mM glucose, 1 mM glutamine, 1% pen‐strep, and 1% FBS for 24 h. Samples of medium were collected before and after the incubation period and subsequently analyzed by mass spectrometry. After the 24 h incubation period, cells were washed twice with cold PBS and the intracellular metabolites extracted with 70% ethanol (Amaral et al., 2014). Astrocyte cultures were also incubated for 24 h in 2 mL DMEM containing 2 mM d‐glucose, 1 mM glutamine, 1% pen‐strep, and 1% FBS in order to determine glucose consumption and lactate production rates after 24 h. Experiments were performed on 9–12 samples, which derived from a minimum of three independently generated cultures.

### Immunocytochemistry

OPCs were seeded on PLL‐coated glass coverslips on 24 well plates (7 × 10^4^ cells/well) and fixed after 5 days of differentiation with 4% paraformaldehyde. Cells were stained with an anti‐O4 mouse monoclonal antibody (1:200; Sigma, Dorset, UK), anti‐myelin basic protein (MBP) rat polyclonal antibody (1:300; Merck Millipore, Hertfordshire, UK), and anti‐GFAP rabbit polyclonal antibody (1/500; Dako, Glostrup, Denmark). Secondary antibodies conjugated with Alexa Fluor 488 and Alexa Fluor 555 were used to visualize positive cells (1:500; Life Technologies, Paisley, UK). Following immunocytochemistry, cells were mounted with Prolong gold antifade mounting medium (Life Technologies, Paisley, UK). To assess purity and differentiation of oligodendroglia lineage cells in the cultures, the percentage of O4‐, MBP‐, and GFAP‐positive cells relative to >100 4′,6‐diamidino‐2‐phenylindole (DAPI)‐stained nuclei per experiment in randomly selected eye fields was determined. Cells were visualized and digitized at ambient temperature on an LSM 700 confocal microscope (Zeiss, Thornwood, NJ) at 20× magnification using Zen Application software (Zeiss).

### Assessment of Cell Viability

To assess cell viability under the different experimental conditions, the Dead End™ Fluorometric Terminal deoxynucleotidyl transferase dUTP nick end labeling (TUNEL) System Kit (Promega, Madison, WI) was used according to the manufacturer's instructions. Images of the stained cells were obtained using an INCell Analyzer 2200 Imaging System (GE Healthcare Life Sciences) and processed using the ImageJ software. Ten random fields were acquired per condition using a magnification of 20×. Image analysis and quantification was performed on *CellProfiler, a* cell image analysis software developed by the BROAD Institute. The number of DAPI‐positive nuclei (total number of cells) and the number of TUNEL‐positive nuclei were automatically counted, and the results presented as percentage of apoptotic nuclei of all DAPI‐positive nuclei.

### Glucose and Lactate Analyses

Glucose and lactate levels in the cell culture medium were analyzed at the Core Biochemical Assay Laboratory, Clinical Biochemistry, Addenbrooke's Hospital using automated assays on a Siemens Dimension RxL analyser. The rate of glucose and lactate net change relative to cells over time (µmol/10^6^ cells/24 h) was calculated by subtracting the value measured at the end of the experiment (*T* = 24 h) from the one measured in a sample of medium collected at the onset of the incubation, and dividing the resulting value by the amount of cells in each experiment, multiplied by the experimental volume (2 mL). For oligodendrocyte cultures, the cell number considered was the cell number at plating since these cells do not proliferate. For astrocyte cultures (which proliferate to some extent), the cell number considered was determined at the end of the experiment, after collection of cells from two sample wells using trypsin‐EDTA.

### High Performance Liquid Chromatography

High performance liquid chromatography (HPLC) was used to quantify the total amounts of amino acids in samples of cell extracts and medium. Samples were lyophilized and resuspended in 0.01M HCl and subsequently derivatized with *o*‐phtaldialdehyde (Geddes and Wood, 1984) using an automated method prior to injection into the HPLC column. The amino acid components were separated with a ZORBAX SB‐C18 (4.6 × 150 mm, 3.55 μm) column from Agilent Technologies (Palo Alto, CA). As eluents, a mixture of 50 mM sodium phosphate buffer (pH 5.9) with 2.5% tetrahydrofurane and a mixture of methanol (98.75%) with tetrahydrofurane (1.25%) were used. The samples were analyzed using a Hewlett Packard 1100 System (Agilent Technologies, Palo Alto, CA) with fluorescence detection. Amino acid concentrations were determined by comparison to a calibration curve of standard solutions of amino acids run after every 12 samples (Amaral et al., 2014).

### Gas Chromatography–Mass Spectrometry

For analysis of percent enrichment of ^13^C in lactate, amino acids (alanine, aspartate, glutamate, and glutamine) and TCA cycle intermediates (citrate and malate) after incubation with different ^13^C‐labelled substrates, cell extracts, and samples of medium were lyophilized and resuspended in 0.01M HCl. To move the metabolites of interest into the organic phase in their acid form, the pH was adjusted to pH < 2 with 6M HCl. Samples were dried under atmospheric air (50°C), and metabolites were derivatized with MTBSTFA in the presence of 1% *t*‐BDMS‐Cl (Mawhinney et al., 1986). The protocol used for analysis of the glycolytic intermediates phosphoenolpyruvate (PEP) and 3‐phosphoglycerate (3PG) was based on the protocol reported by Hofmann et al. (2008). Derivatization was performed using a mixture of MSTFA + 1% trimethylchlorosilane and acetonitrile. The samples were analyzed on an Agilent 6890 gas chromatograph connected to an Agilent 5975B mass spectrometer (Agilent Technologies, Palo Alto, CA). The parent ion (*M*) and atom percent excess for one ^13^C atom (*M*+1) values for 3PG, PEP, alanine, aspartate, lactate, citrate, and glutamate were calculated from the gas chromatography‐mass spectrometry (GC–MS) data using the MassHunter software supplied by Agilent (Agilent Technologies, Palo Alto, CA) and correcting for the naturally abundant ^13^C using nonenriched standards (Biemann, 1962).

### ^13^C And ^1^H NMR Spectroscopy

^13^C NMR spectroscopy was used to identify the synthesis of particular labelled isotopologues from [1,2‐^13^C]glucose metabolism due to the ability of this technique to distinguish between the different carbon positions that are labelled in one molecule. For example, ^13^C NMR spectroscopy enabled to distinguish between the presence of [2,3‐^13^C]glutamate (synthesized via pyruvate carboxylation) and [1,2‐^13^C]glutamate (synthesized via PDH; see below for further details). In contrast, GC–MS is a more sensitive method that provides information about ^13^C enrichment above natural abundance but lacks the specificity of NMR spectroscopy, as it does not provide information about the location of the ^13^C label. GC–MS, as applied in this study, only enables to distinguish between species that have a different number of ^13^C‐labelled carbons. All NMR samples were analyzed using a QCI CryoProbe™ 600 MHz (for proton) ultrashielded Plus magnet (Bruker BioSpin GmbH, Reinstetten, Germany). ^1^H NMR spectra were acquired using a pulse angle of 90°, 12 kHz spectral width with 66 data points, acquisition time of 2.66 s, relaxation delay of 10 s and 128 scans. These spectra were used to quantify the amount of glutamate for correction of natural abundance of [4‐^13^C]glutamate (to be used in the calculation of contribution of the PPP to [4‐^13^C]glutamate synthesis). Proton decoupled ^13^C NMR spectra were obtained on the same instrument using a 30° pulse angle and 30 kHz spectral width with 98,000 data points using an acquisition time of 1.65 s and a relaxation delay of 0.5 s. The number of scans needed to obtain an appropriate signal to noise ratio was 210,000. TopSpin™ 3.0 software (Bruker BioSpin GmbH, Reinstetten, Germany) was used for acquisition, integration, and quantification. Relevant peaks in the spectra were assigned and quantified from the integrals of the peaks using ethylene glycol as an internal standard with known amount of ^13^C. Corrections for natural abundance as well as nuclear Overhauser enhancement and relaxation effects, relative to the internal standard, were applied to all relevant integrals from ^13^C spectra.

### Statistical Analysis

Statistical analysis was conducted using unpaired two‐tailed student's *t*‐tests (confidence interval = 95%).

## Results

### Characterization of Late Differentiation‐Stage Oligodendrocyte Lineage Cells *In Vitro*

To study metabolic reactions in mature oligodendroglia cultures, highly enriched primary rat OPCs (Fig. 1A) were differentiated in Sato's differentiation medium. At 5 days of differentiation, the cells displayed the characteristically branched morphology of late oligodendrocyte lineage cells with approximately 65% of the cells expressing MBP (Fig. 1B). To investigate the relative activity of different metabolic pathways in mature oligodendrocytes, cells were incubated with one of the following ^13^C‐labelled substrates: [1,6‐^13^C]glucose, [1,2‐^13^C]glucose, [1‐^13^C]lactate, or [1,2‐^13^C]acetate.

### Differentiated Oligodendrocyte Lineage Cells Display a Significant Activity of the PPP

**Figure 2:**
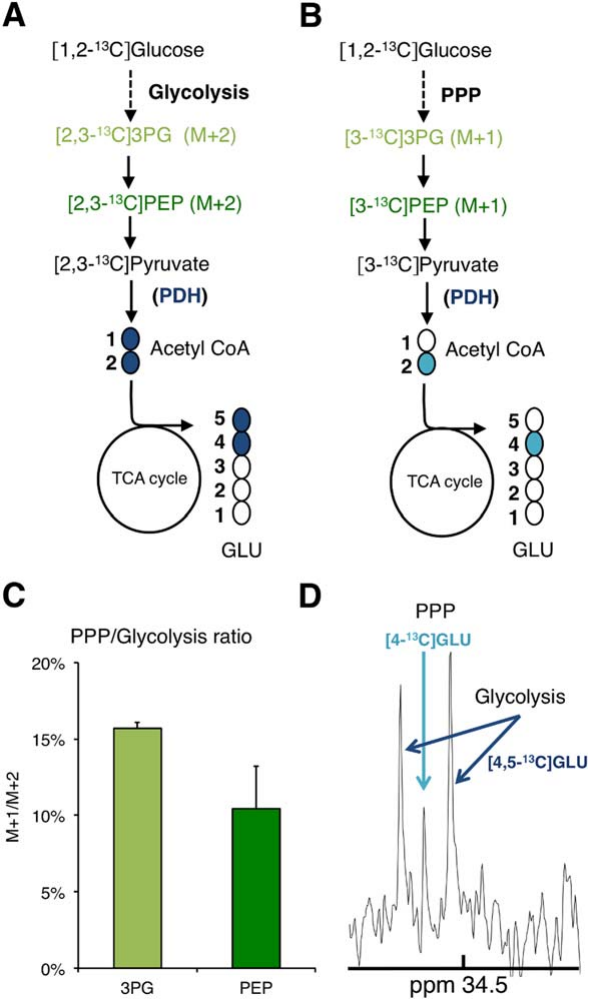
Evidence of pentose phosphate pathway (PPP) activity in mature oligodendrocytes in culture after incubation with [1,2‐^13^C]glucose. Labelling patterns derived from [1,2‐^13^C]glucose metabolism via glycolysis (**A**) and the PPP (**B**). The % enrichment of ^13^C in M+1 and M+2 isotopologues for 3PG and PEP was determined using GC–MS analysis of cell extracts after 24‐h incubation with [1,2‐^13^C]glucose. The ratio shown in (**C**) between M+1 (labelling from the PPP) and M+2 (labelling from glycolysis) enables to estimate the contribution of the PPP versus glycolysis to the formation of the glycolytic intermediates 3PG and PEP in mature oligodendrocyte (mean ± s.e.m.; *n* = 3). (**D**) ^13^C nuclear magnetic resonance spectrum of a cell extract from cultures incubated in medium containing [1,2‐^13^C]glucose for 24 h. The C‐4 region of glutamate at 34.5 ppm (GLU) is shown. The doublet peak represents [4,5‐^13^C]glutamate ([4,5‐^13^C]GLU) which derives from glucose metabolism via glycolysis only. The singlet peak corresponds to [4‐^13^C]glutamate ([4‐^13^C]GLU) which is produced after glucose metabolism via the PPP. Abbreviations: 3PG, 3‐phosphoglycerate; GLU, glutamate; PEP, phosphoenolpyruvate; PDH, pyruvate dehydrogenase; M+1, parent ion with one ^13^C atom; M+2, parent ion with two ^13^C atoms. [Color figure can be viewed in the online issue, which is available at wileyonlinelibrary.com.]

To investigate the relative activity of the glycolytic pathway versus the PPP, [1,2‐^13^C]glucose was added to the medium (Brekke et al., 2014; Dusick et al., 2007). Following 24 h of incubation, cell extracts were collected and analyzed using GC–MS and ^13^C and ^1^H NMR spectroscopy. If [1,2‐^13^C]glucose is metabolized via the glycolytic pathway, [2,3‐^13^C]3PG, [2,3‐^13^C]PEP, and [2,3‐^13^C]pyruvate are formed (Fig. 2A). [2,3‐^13^C]pyruvate can enter the mitochondria to be converted into [1,2‐^13^C]acetyl CoA. Condensation of [1,2‐^13^C]acetyl CoA with unlabelled oxaloacetate leads to the formation of the TCA cycle intermediate [1,2‐^13^C]citrate and then, following several steps, α‐[4,5‐^13^C]ketoglutarate, which is subsequently converted into [4,5‐^13^C]glutamate. [1,2‐^13^C]glucose metabolism via the PPP gives rise to [3‐^13^C]3PG, [3‐^13^C]PEP, [3‐^13^C]pyruvate, and [2‐^13^C]acetyl CoA (Fig. 2B). [2‐^13^C]acetyl CoA can then be converted into [4‐^13^C]glutamate via the TCA cycle (Fig. 2B).

Because PEP and 3PG are localized downstream of the intersection between glycolysis and the PPP, their ^13^C isotopologues can be used to calculate the ratio between glycolysis and the PPP. Analysis of PEP and 3PG enables detection of isotopologues that derive from the re‐entry of metabolic intermediates (fructose‐6P and GA3P) into glycolysis from the PPP. The intermediates of the PPP lose the ^13^C in the C1 position via decarboxylation of [1,2‐^13^C]gluconate‐6‐phosphate by 6‐phosphogluconate dehydrogenase and 1/3 of the molecules will re‐enter glycolysis with only one labelled (M+1) carbon instead of two (M+2)—see Fig. 2 and Brekke et al. (2012) for further details. Based on GC–MS results, the M+2/M+1 ratios of PEP and 3PG were calculated, indicating that 10–15% of glucose is metabolized via the PPP in mature oligodendrocytes (Fig. 2C). Of note, this ratio is likely to underestimate the contribution of the PPP (for details, see Brekke et al., 2012).

An alternative way to investigate the relative contribution of glycolysis and PPP to glucose metabolism is to apply ^13^C NMR spectroscopy to differentiate between [4,5‐^13^C] and [4‐^13^C]glutamate (Fig. 2D). In the ^13^C NMR spectrum, glycolysis‐derived [4,5‐^13^C]glutamate is represented as a doublet in the C‐4 region of glutamate (34.5 ppm), whereas PPP‐derived [4‐^13^C]glutamate is represented as a singlet. The contribution of glucose metabolized via the PPP relative to glucose metabolized via glycolysis to the total glutamate synthesis was calculated by dividing the area of the [4‐^13^C]glutamate peak (after correction for natural abundance using ^1^H NMR spectroscopy) by the area of the doublet peak [4,5‐^13^C]glutamate. We found that 10 ± 0.2% (mean ± s.e.m.; *n* = 3) of the total glutamate comes from glucose metabolized in the PPP. Our results confirm that the PPP is active in oligodendrocytes and that it contributes to glutamate synthesis.

### Evidence for PDH and Mitochondrial Activity in Oligodendrocytes

**Figure 3:**
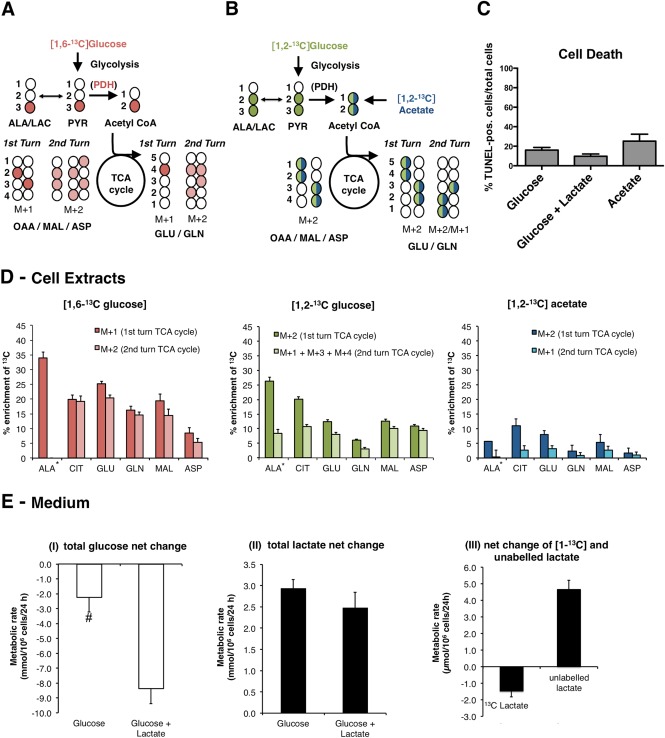
Evidence of high TCA cycle activity and acetate metabolism in mature oligodendrocytes in culture. Oligodendrocytes were differentiated for 5 days and incubated for 24 h in medium containing [1,6‐^13^C]glucose (**A**), [1,2‐^13^C]glucose (**B**), or [1,2‐^13^C]acetate (B), followed by GC–MS analysis of samples of cell culture medium and cell extracts. A and B describe the labelling patterns deriving from the metabolism of these ^13^C‐labelled substrates. The isotopologues formed in the second turn of the TCA cycle result from condensation of labelled oxaloacetate (OAA) with labelled or unlabelled acetyl CoA. (**C**) Quantification of TUNEL positive/total cell number (given by DAPI staining) in cells incubated with glucose alone (mean ± s.e.m., *n* = 12), glucose and lactate (mean ± s.e.m., *n* = 10), or acetate (mean ± s.e.m., *n* = 8); (**D**) % enrichment of ^13^C in intracellular alanine and TCA cycle‐related metabolites derived from each of the substrates (mean ± s.e.m., *n* = 8 for [1,6‐^13^C]glucose, mean ± s.e.m., *n* = 6 for [1,2‐^13^C]glucose and mean ± s.e.m., *n* = 12 for [1,2‐^13^C]acetate). (**E**) Glucose (I) and lactate (II) net change in the medium in experiments performed in the presence of glucose alone or glucose + [1‐^13^C]lactate. For the experiment where [1‐^13^C]lactate was used, the net change of ^13^C‐labelled and unlabelled lactate is shown (III) (mean ± s.e.m., *n* = 9). #—significantly different from the glucose + [1‐^13^C]lactate group (*P* < 0.05, Student's *t*‐test). Abbreviations: ALA, alanine; ASP, aspartate; CIT, citrate; GLN, glutamine; GLU, glutamate; MAL, malate; PYR, pyruvate; M+1, parent ion with one ^13^C atom; M+2, parent ion with two ^13^C atoms; M+3, parent ion with three ^13^C atoms; M+4, parent ion with four ^13^C atoms. *The enrichment detected in alanine derives directly from ^13^C‐labelled pyruvate and not from the TCA cycle when [1,6‐^13^C]glucose is the precursor. When [1,2‐^13^C]glucose is used, M+2 is not derived from the TCA cycle but M+1 alanine is. When [1,2‐^13^C]acetate is in the medium, both alanine isotopologues are derived from the TCA cycle. [Color figure can be viewed in the online issue, which is available at wileyonlinelibrary.com.]

To investigate the extent of oxidative metabolism in oligodendrocytes, cells were incubated with ^13^C‐labelled glucose or [1,2‐^13^C]acetate for 24 h (for labelling patterns, see Fig. 3A, B). We confirmed that after 24 h, all [1,6‐^13^C]glucose‐derived metabolites, except for glutamine, reached a steady‐state of labelling (Supp. Info. Fig. 1). Hence, we decided to investigate labelling patterns in cell lysates after 24 h in all subsequent experiments. Assessing cell viability using TUNEL staining showed no differences between the various experimental conditions (Fig. 3C). Metabolism of [1,6‐^13^C]glucose yields two molecules of [3‐^13^C]alanine and [3‐^13^C]pyruvate. The latter is then converted into [2‐^13^C]acetyl CoA, which can condense with nonlabelled oxaloacetate to form monolabelled (M+1) compounds in the first turn of the TCA cycle (described in Fig. 3A). [2‐^13^C]acetyl CoA can also condense with labelled oxaloacetate and give rise to the formation of double‐labelled (M+2) compounds in a combination of the first and second turn of the cycle (Fig. 3A). GC–MS analysis of cell extracts incubated with [1,6‐^13^C]glucose (Fig. 3D), showed that mitochondrial metabolism was prominent in mature oligodendrocytes. TCA cycle intermediates and amino acids were highly enriched ranging from 15 to 25% in citrate, malate, glutamate, and glutamine and 10% in aspartate (Fig. 3D). Moreover, a substantial enrichment was also observed with M+2 isotopologues and even M+3 (data not shown), typical of the second and third turns of the TCA cycle (see Fig. 3D). However, the second turn of the TCA cycle is underestimated since labelled oxaloacetate can condense with unlabelled acetyl CoA and give rise to single labelled (M+1) compounds in the second turn. Second turn isotopologues were also observed when [1,2‐^13^C]glucose was used as substrate (Fig. 3D). It is important to note that only half of the pyruvate molecules are labelled from [1,2‐^13^C]glucose and, therefore, the maximum enrichment levels will be half of those obtained from [1,6‐^13^C]glucose. Alanine is obtained from pyruvate transamination and is generally considered to be a metabolite related to glycolysis. The expected isotopologue of [1,2‐^13^C]glucose is [1,2‐^13^C]alanine. However, we observed M+1 alanine in addition to M+2‐labelled alanine (Fig. 3D). This is evidence for the presence of partial pyruvate recycling, which can be performed via decarboxylation of malate or oxaloacetate into pyruvate (Sonnewald, 2014).

### Acetate Metabolism in Oligodendrocytes

Conversion of acetate into acetyl CoA is known to take place in astrocytes, but not in neurons (Sonnewald and Rae, 2010). To investigate whether oligodendrocytes are able to convert acetate into acetyl CoA and oxidize it in the TCA cycle, cells were incubated with [1,2‐^13^C]acetate for 24 h and the extra‐ and intracellular metabolites were analyzed by GC–MS (Fig. 3B, D). Both [1,2‐^13^C]acetate and [1,2‐^13^C]glucose identically yield [1,2‐^13^C]acetyl CoA (Fig. 3B). Overall, the extent of labelling from [1,2‐^13^C]acetate was much lower than that from [1,2‐^13^C]glucose. Enrichment of the most abundant metabolites citrate and glutamate from [1,2‐^13^C]acetate was approximately half of enrichment obtained from [1,2‐^13^C]glucose; the less‐abundant metabolites, aspartate and glutamine, were poorly enriched. The abundance of M+1 isotopologues (typical of the second turn of the TCA cycle) derived from [1,2‐^13^C]acetate was very low (Fig. 3D), suggesting that acetate‐derived acetyl CoA is metabolized in a different compartment than pyruvate‐derived acetyl CoA. Alanine enrichment from [1,2‐^13^C]acetate (albeit at low levels), confirms that oligodendrocytes have active partial pyruvate recycling, as observed in cells incubated with [1,2‐^13^C]glucose. In the medium, labelling from [1,2‐^13^C]acetate was only detectable in citrate, in which M+2 amounted to 10 ± 2% (mean ± s.e.m., *n* = 12).

### Glucose Consumption and Release of Metabolites to the Culture Medium

Analysis of glucose consumption and lactate release rates (Fig. 3E‐I, II) indicates that oligodendrocytes metabolize glucose to an extent comparable to astrocytes (3.2 ± 0.06 µmol/10^6^ cells/24 h; mean ± s.e.m.; *n* = 6). However, oligodendrocytes release less lactate than astrocytes (5.3 ± 0.16 µmol/10^6^ cells/24 h; mean ± s.e.m.; *n* = 6). Although glucose consumption in the presence of 4 mM [1‐^13^C]lactate (Fig. 3E‐I) was increased, the net release of total lactate remained unchanged (Fig. 3E‐II). Due to the presence of [1‐^13^C]lactate in the culture medium, it was possible to distinguish between the uptake of [1‐^13^C]lactate and the release of endogenous (unlabelled) lactate (Fig. 3E‐III). This analysis showed that the presence of lactate in the medium increased the release of endogenous lactate (Fig. 3E‐III). The release of unlabelled lactate was at least 3‐fold larger than the amount of [1‐^13^C]lactate consumed. By comparing lactate release rates and glucose consumption rates, it was possible to estimate the fraction of glucose metabolized to lactate, versus the oxidation of pyruvate in the mitochondria. In oligodendrocytes cultured with glucose alone, the ratio of lactate production to pyruvate oxidation was 60:40 (and 80:20 in astrocytes). In the presence of lactate, the ratio changed to 30:70, indicating increased mitochondrial activity. Figure 3C shows that the viability of cells was not affected by the presence of lactate.

Despite the substantial % enrichment of ^13^C in the aforementioned amino acids observed in the cell extracts, only negligible release of alanine and glutamine was detected (data not shown).

### Pyruvate Carboxylation in Oligodendrocytes

**Figure 4:**
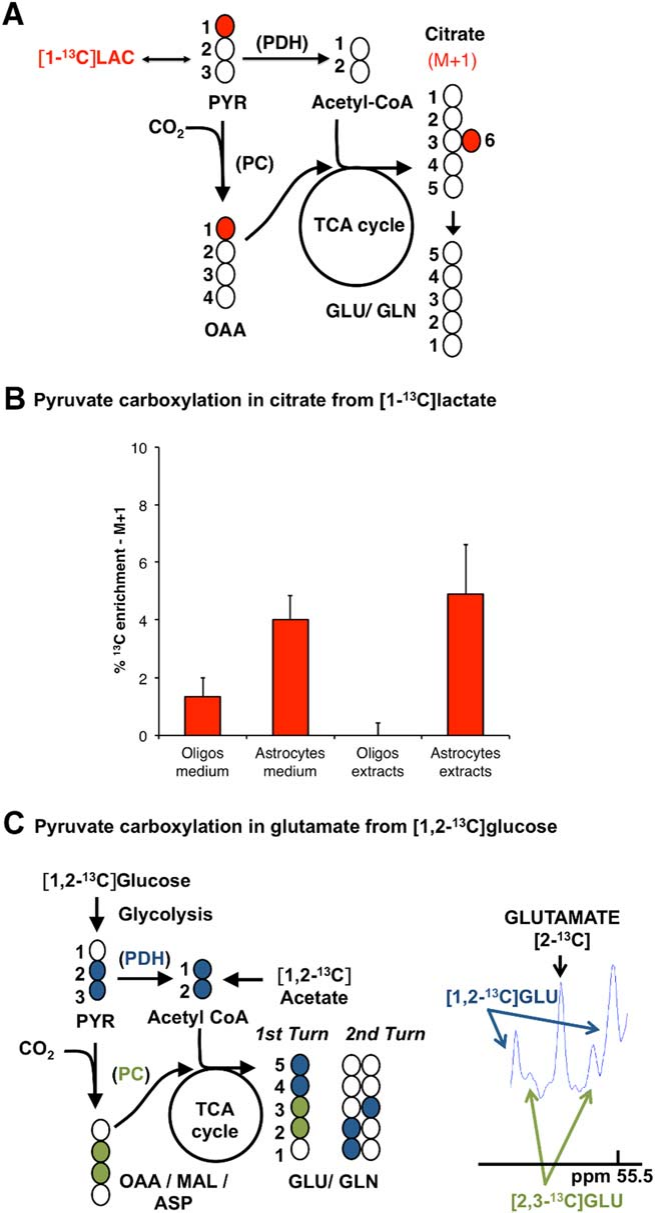
[1‐^13^C]lactate metabolism and evidence for pyruvate carboxylation in oligodendrocytes. (**A**) Labelling patterns resulting from the metabolism of [1‐^13^C]lactate (^13^C atoms are identified as filled circles): [1‐^13^C]lactate (LAC) is converted into [1‐^13^C]pyruvate, which can be converted into acetyl CoA via PDH or into [1‐^13^C]oxaloacetate (OAA) via pyruvate carboxylase (PC). The first carbon of pyruvate is lost in its conversion to acetyl CoA via PDH. Therefore, the ^13^C label can only be observed downstream of pyruvate if PC is active. [1‐^13^C]oxaloacetate can condense with acetyl CoA to form [6‐^13^C]citrate, which also leads subsequently to the formation of unlabelled α‐ketoglutarate by loss of carbon 6. (**B**) The % enrichment of ^13^C in citrate indicates the contribution of pyruvate carboxylation to citrate formation from [1‐^13^C]lactate. The % of M+1 citrate was assessed using GC–MS in samples of cell extracts and culture medium from differentiated oligodendrocytes and also in cultures of cortical astrocytes after 24 h of incubation with [1‐^13^C]lactate (oligodendrocytes—mean ± s.e.m., *n* = 9; and astrocytes—mean ± s.e.m., *n* = 6). (**C**) Contribution of pyruvate carboxylation to glutamate synthesis from [1,2‐^13^C]glucose. Oligodendrocytes were incubated in medium containing [1,2‐^13^C]glucose for 24 h, extracted, and analyzed using ^13^C‐magnetic resonance spectroscopy for the presence of isotopologues of glutamate formed via pyruvate carboxylation. The C‐2 region of glutamate (GLU) at 55.5 ppm is shown. The doublet representing [2,3‐^13^C]glutamate is formed via pyruvate carboxylation and the doublet representing [1,2‐^13^C]glutamate is formed via pyruvate dehydrogenation. Abbreviations: ASP, aspartate; GLN, glutamine; GLU, glutamate; MAL, malate; OAA, oxaloacetate; PC, pyruvate carboxylase; PDH, pyruvate dehydrogenase; PYR, pyruvate. [Color figure can be viewed in the online issue, which is available at wileyonlinelibrary.com.]

Pyruvate carboxylation is an important anaplerotic pathway known to operate in astrocytes but not in neurons (McKenna et al., 2012). The presence of this pathway was probed in mature oligodendrocytes using either [1,2‐^13^C]glucose or [1‐^13^C]lactate (Fig. 4). [1‐^13^C]lactate is a valuable substrate for this purpose since only via pyruvate carboxylation the ^13^C label from [1‐^13^C]lactate can be found in the TCA cycle intermediate citrate (PDH removes carbon number 1 from pyruvate generating unlabelled acetyl CoA, and therefore, the ^13^C label is lost via PDH; Fig. 4A). In order to evaluate the significance of pyruvate carboxylation in oligodendrocytes, also astrocytes (the cells known to carboxylate pyruvate in the brain) were incubated with [1‐^13^C]lactate. Both of these cell types were shown to oxidize lactate to a large extent in culture (Sanchez‐Abarca et al., 2001). [1‐^13^C]Pyruvate carboxylation was apparent in the labelling of citrate in the medium in both cell types (Fig. 4B). However, in the cell extracts, this was only evident in astrocytes. A possible explanation for the absence of intracellular citrate enrichment in oligodendrocytes is a potential compartmentation of pyruvate metabolism, which has also been shown for astrocytes and neurons (Bak et al., 2007; Bakken et al., 1997).

To further investigate the presence of pyruvate carboxylation, oligodendrocytes were also incubated with medium containing [1,2‐^13^C]glucose. The C‐2 region of glutamate (around 55.5 ppm) in the ^13^C NMR spectrum of cell extracts was analyzed to confirm the presence of pyruvate carboxylation (Fig. 4C). From the scheme depicted in Fig. 4C, it emerges that only pyruvate carboxylation will lead to the formation of [2,3‐^13^C]glutamate, whereas [1,2‐^13^C]glutamate (and [3‐^13^C]glutamate, which is not shown) is formed from pyruvate dehydrogenation (and pyruvate carboxylation, if the label stays in the cycle for an additional turn). The spectrum shown in Fig. 4C, which reflects the presence of [2,3‐^13^C]glutamate, indicates that oligodendrocytes carboxylate pyruvate.

## Discussion

Although oligodendrocytes make up a significant proportion of brain cells, their metabolic properties remain largely unknown. In a recent review, we have called for a systematic study of metabolic pathways in oligodendrocytes (Amaral et al., 2013). In this work, we study aspects of glucose, lactate, and acetate metabolism in oligodendrocytes, and specifically address the activity of the PPP and whether oligodendrocytes are able to conduct anaplerotic and cataplerotic reactions based on carboxylation of pyruvate and decarboxylation of malate or oxaloacetate.

Primary rat OPCs were purified from perinatal rat‐mixed glial cultures (McCarthy and de Vellis, 1980). This approach results in high yields of relatively pure (>93%) cultures. Culture of the O4+, A2B5+, O1‐, CNP‐, and MBP‐ OPCs in Sato's medium triggers a highly predictable series of morphological and transcriptional events and results in the formation of mature oligodendrocytes with complex‐branched processes and membrane sheets, which express late stage markers, including MBP. Although a significant proportion of cells (approximately 60%) reach a mature oligodendrocyte stage, the cultures also include late stage progenitors. Genomic studies comparing acutely isolated cells at distinct developmental stages with the culture system used in this study confirmed that primary OPC cultures faithfully represent their *in vivo* counterparts (Dugas et al., 2006).

Our results demonstrate that oligodendrocytes have extensive glucose‐derived metabolism. In fact, we found that the rate of glucose consumption in oligodendrocytes is comparable to the one in astrocytes. Although Sanchez‐Abarca et al. (2001) previously concluded that oligodendrocytes use more glucose than astrocytes, they also showed that oligodendrocytes metabolize a larger proportion of glucose via PDH than astrocytes. We obtained a similar result by comparing the ratios of lactate release to glucose consumption in both cell types. Furthermore, we observed that both glucose consumption and the proportion of glucose‐derived pyruvate metabolized in the mitochondria were increased in the presence of exogenous lactate. This suggests that lactate may act as metabolic activator in oligodendrocytes, fitting with the concept that lactate can act as signalling molecule (Rinholm and Bergersen, 2014). Furthermore, our results confirm that oligodendrocytes are able to release substantial amounts of lactate *in vitro* as has been reported *in vivo* by Funfschilling et al. (2012). Also, it is possible that, at least, a proportion of the [1‐^13^C]lactate taken up was oxidized for lipogenesis, as proposed by (Sanchez‐Abarca et al., 2001). In spite of the presence of 4‐mM exogenous lactate, a net production of lactate was observed indicating that glycolysis in oligodendrocytes is not inhibited by extracellular lactate.

The PPP is a glucose shunt, which is thought to be active in neurons and astrocytes (Almeida et al., 2002; Amaral et al., 2010; Bolanos and Almeida, 2010; Brekke et al., 2012; Garcia‐Nogales et al., 2003). Using ^14^C tracing techniques, Edmond et al. (1987) showed active PPP in oligodendrocyte lineage cells. Sanchez‐Abarca et al. (2001) reported PPP activity in immature OPC cultures as being 2‐fold higher than in astrocytes and 4‐fold higher than in neurons. Our approach measured the contribution of the PPP to the synthesis of glycolytic intermediates and glutamate synthesis based on ^13^C‐tracing techniques. In our study, the labelling of the glycolytic intermediates PEP and 3PG indicate that mature oligodendrocytes use approximately 10–15% of glucose in the PPP compared with glycolysis. These values are in the range of previously published data for cortical astrocyte cultures in a metabolic modelling study also using ^13^C‐labelled substrates (Amaral et al., 2011a), which contrasts with the report by Sanchez‐Abarca et al. (2001). It is possible that our cultures contain more mature oligodendrocytes than those used by Sanchez‐Abarca et al., and therefore, have a lower PPP activity, more closely resembling the rate in astrocytes. On the basis of ^14^C tracing experiments, Sykes et al. (1986) reported that, although the PPP oxidizes only <3% of the glucose consumed, it produces more CO_2_ than the TCA cycle in primary oligodendrocyte lineage cells, due to its close link to de novo synthesis of fatty acids and cholesterol. Since we did not measure the contribution of glucose to lipid synthesis, it is possible that our approach underestimated the total use of glucose via the PPP.

For the first time, our experiments demonstrate that pyruvate generated from glucose via the PPP contributes to the synthesis of acetyl CoA for oxidation and generation of metabolites in the TCA cycle in oligodendrocytes. We found that approximately 10% of the glutamate produced derives from glucose metabolized in the PPP. Estimation of the PPP activity on the basis of glutamate isotopomers indicated that the PPP accounted for approximately 6% of glucose metabolism in cortical neurons and approximately 4% in cerebellar neurons (Brekke et al., 2012). Similar to reports in neurons (Brekke et al., 2012), we found that oligodendrocytes incorporate ^13^C label in glutamate produced by [1,2‐^13^C]glucose metabolism via the PPP.

The PPP may also play a role in diseases that affect oligodendrocytes. For example, increased activity of the transaldolase, an enzyme which forms part of the nonoxidative branch of the PPP and is involved in lipid and nucleotide synthesis, has been reported in oligodendrocytes in brains of MS patients, compared with healthy controls (Banki et al., 1994). NADPH produced in the PPP is fundamentally important for the synthesis of glutathione, which is thought to protect myelin sheaths from oxidative stress. Banki and colleagues proposed that autoantibodies against transaldolase found in MS patients, may cause destruction of oligodendrocytes via depletion of transaldolase. Altered PPP activity was also reported in a study of patients that suffered a traumatic brain injury (TBI; Dusick et al., 2007). It is possible that, at least, a proportion of the PPP alterations observed in the study by Dusick et al. (2007) could be linked to the extensive demyelination that is thought to occur in TBI patients (Armstrong et al., in press).

Using different forms of ^13^C‐labelled glucose, we demonstrate that our cultures also exhibit a high rate of mitochondrial metabolism, as previously suggested by Sanchez‐Abarca et al. (2001). Whereas functional mitochondria seem to be important for OPC differentiation (Schoenfeld et al., 2010; Ziabreva et al., 2010), it was recently proposed that myelinating oligodendrocytes are not dependent on mitochondrial activity *in vivo* (Funfschilling et al., 2012). The most likely explanation for the discrepancy between the high mitochondrial demands of our cultures and the findings *in vivo* is the significant presence of premyelinating (MBP‐negative) OPCs that have not yet reached fully mature stages *in vitro*. Our data thus may reflect the prominent role of oxidative metabolism at the late stages of OPC differentiation, including the premyelinating and early myelinating stages.

Another important question with respect to mitochondrial metabolism in oligodendrocytes is whether they can replenish TCA cycle intermediates via anaplerosis. It is well established that neurons depend on astrocytes for replenishing their TCA cycle intermediates. As neurons cannot carboxylate pyruvate, external TCA cycle intermediates are required for the synthesis of amino acid neurotransmitters (McKenna et al., 2012). Whether and to which extent oligodendrocytes are self‐sufficient with respect to the production of anaplerotic substrates remained unknown. By incubating cells with [1,2‐^13^C]glucose, we found that pyruvate carboxylation indeed takes place in oligodendrocytes as shown by the production of [2,3‐^13^C]glutamate. This was further confirmed by label incorporation in citrate in the medium of cells incubated with [1‐^13^C]lactate, which is only possible via pyruvate carboxylation. Comparable label incorporation from [1‐^13^C]lactate was found in citrate in the medium of astrocyte cultures. This suggests that lactate is metabolized in a similar way in the TCA cycle of both cell types. However, it is possible that a fraction of the lactate taken up by our cultures was used for lipogenesis (Sanchez‐Abarca et al., 2001). It must also be noted that the overall contribution of pyruvate carboxylation to oligodendrocyte and astrocyte metabolism is underestimated in the experiments based on [1‐^13^C]lactate metabolism because [1‐^13^C]pyruvate derived from [1‐^13^C]lactate competes with unlabelled pyruvate derived from glucose, which is also present in the incubation medium.

Pyruvate can be carboxylated to oxaloacetate by PC or to malate and NADP+ by malic enzyme. Whether PC or malic enzyme is responsible for pyruvate carboxylation in oligodendrocytes remains to be established. Murin et al. (2009) reported PC expression in cultured oligodendroglia. Whether oligodendrocytes express malic enzyme remains unknown. In neurons and astrocytes, malic enzyme only contributes to pyruvate production (McKenna et al., 2000, 1995). However, PC is thought to be the most important anaplerotic enzyme in the brain (Patel, 1974). Irrespective of which enzyme is responsible for pyruvate carboxylation, the detection of carboxylation (and thus anaplerosis) has consequences for oligodendrocyte metabolism. If oligodendrocytes (similarly to neurons) were not capable of anaplerosis, they would depend on the provision of glutamine by astrocytes, which have a net production of glutamine via pyruvate carboxylation (Gamberino et al., 1997; Waagepetersen et al., 2001).

An alternative route to replenish the TCA cycle in oligodendrocytes is to use aspartate, liberated from *N*‐acetyl aspartate (NAA), which in turn is supplied by neurons (Moffett et al., 2007). However, aspartate released by NAA hydrolysis in oligodendrocytes could potentially be sent back to neurons, thus avoiding the depletion of anaplerotic substrates in neurons (NAA synthesis in neurons is dependent on glutamine entry from astrocytes). Our results indicate that oligodendrocytes are capable of anaplerosis, which suggests that they are potentially independent of astrocytic pyruvate carboxylation. Whether the level of anaplerosis in oligodendrocytes is sufficient to meet their entire requirements is not known at present. It is also unclear whether aspartate is shuttled back to neurons or whether it is metabolized in oligodendrocytes. Both possibilities have previously been suggested but evidence is lacking (Baslow and Guilfoyle, 2006; Moffett et al., 2007).

Acetyl CoA is an essential molecule in the TCA cycle. Most acetyl CoA derives from pyruvate via PDH. However, oligodendrocytes are known to express the enzyme aspartoacylase, which catalyzes the hydrolysis of NAA into aspartate and acetate (Moffett et al., 2011). NAA‐derived acetate significantly contributes to myelin lipid synthesis in the CNS (Chakraborty et al., 2001) and is also thought to support oxidative metabolism during myelination (Francis et al., 2012). Furthermore, oligodendrocytes express acetyl CoA synthetase‐1, which catalyzes the synthesis of acetyl coenzyme A from acetate and coenzyme A, indicating that acetate may contribute to lipid synthesis, especially during postnatal brain development (Ariyannur et al., 2010). So far only astrocytes were shown to convert acetate into acetyl CoA whereas neurons do not seem able to do so (Muir et al., 1986; Sonnewald et al., 1993a). Consequently, acetate has been used extensively to assess astrocyte metabolism in the context of astrocytic‐neuronal interactions *in vivo* (e.g., Melo et al., 2005; Morken et al., 2014; Nilsen et al., 2014), in neurospheres (Sa Santos et al., 2011), and in culture (Sonnewald et al., 1993a). However, potential metabolic contributions from oligodendrocytes were not considered in these studies. This work demonstrates that mature oligodendrocyte cultures are able to convert acetate into acetyl CoA and oxidize it in the mitochondria as shown by the incorporation of ^13^C label from [1,2‐^13^C]acetate into the TCA cycle intermediates malate and citrate and the amino acids glutamate and glutamine. Labelling from [1,2‐^13^C]acetate was not as pronounced as labelling from [1,2‐^13^C]glucose but, nevertheless, significant and comparable to that observed in astrocytes (data not shown). However, astrocytes label glutamine extensively from ^13^C‐labelled acetate (Hassel et al., 1995), whereas oligodendrocytes do not. Surprisingly, we found that alanine was also labelled from [1,2‐^13^C]acetate, and that alanine M+1 enrichment was detected in oligodendrocytes incubated with [1,2‐^13^C]glucose. Both isotopologues of alanine could not have been produced without the participation of the TCA cycle and malic enzyme or pyruvate kinase and phosphoenolpyruvate carboxykinase (Cruz et al., 1998). This indicates that pyruvate recycling, a catabolic pathway (Amaral et al., 2011b; Cerdan et al., 1990; Haberg et al., 1998; Kunnecke et al., 1993; Olstad et al., 2007), is not only active in astrocytes and neurons but also in oligodendrocytes.

## Conclusion

**Figure 5:**
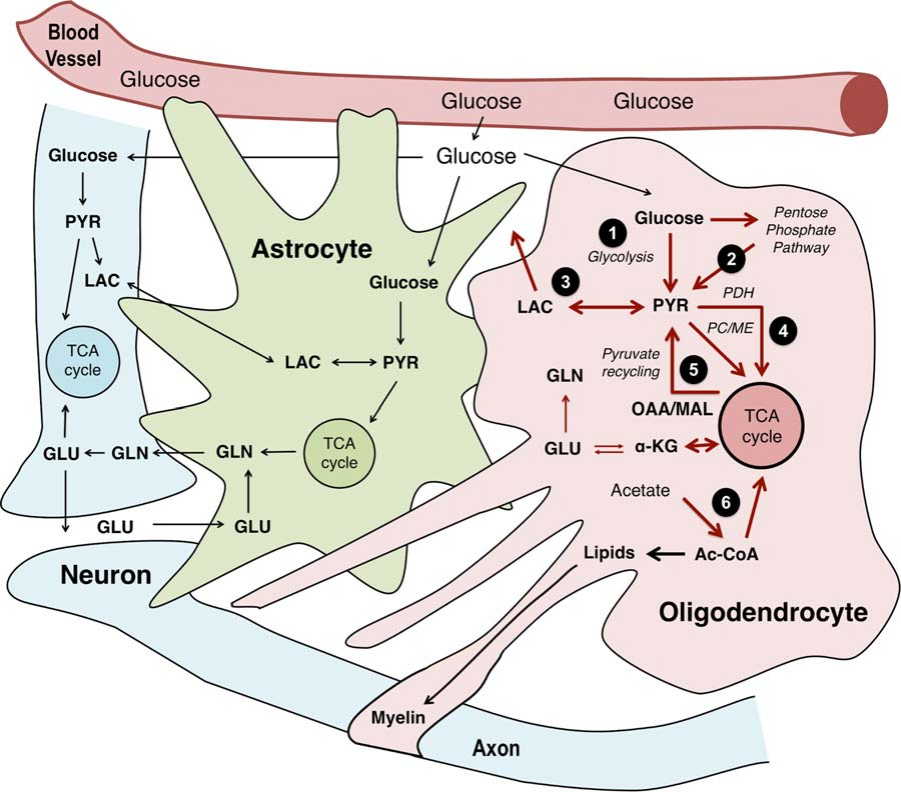
Integration of metabolic pathways operating in oligodendrocytes in the context of metabolic interactions with astrocytes and neurons/axons. The pathways investigated in this study are highlighted in red in the oligodendrocyte: glucose is taken up and subsequently metabolized either via glycolysis only (1) or also via the pentose phosphate pathway (2); the resulting pyruvate (PYR) produced can be reduced to lactate (LAC) (3) which can be released and taken up by cells with lower lactate concentration. Moreover, pyruvate can be carboxylated via PC or malic enzyme (ME) into oxaloacetate (OAA) or malate (MAL) or enter the TCA cycle after being converted to acetyl CoA (Ac‐CoA) via PDH (4). The TCA cycle intermediate α‐ketoglutarate (α‐KG) gives rise to glutamate (GLU) and, subsequently, glutamine (GLN), but none of these aminoacids appear to be significantly released. Pyruvate can be further completely oxidized if it is decarboxylated via ME, a pathway called pyruvate recycling (5), which also seems to be present in this cell type. Oligodendrocytes can also metabolize acetate into acetyl CoA (6) that can be then incorporated into lipids or oxidized in the TCA cycle. [Color figure can be viewed in the online issue, which is available at wileyonlinelibrary.com.]

Our results show that oligodendrocyte lineage cells at late stages of differentiation are metabolically active cells and have distinct metabolic properties. We found that the cells were able to conduct all the metabolic functions that were investigated and, therefore, demonstrate a high degree of cellular independence. Figure 5 summarizes the findings of the present series of experiments and integrates our data with the known pathways linking astroglial and neuronal metabolism. Our results indicate that metabolic functions of oligodendroglia need to be considered in studies investigating glucose metabolism in CNS tissue or whole brain studies. This work reinforces the emerging role of oligodendrocyte metabolism with respect to neuronal–glial interactions.

## Supporting information

Supporting Information Figure 1

## References

[glia22900-bib-0001] Almeida A , Delgado‐Esteban M , Bolanos JP , Medina JM. 2002 Oxygen and glucose deprivation induces mitochondrial dysfunction and oxidative stress in neurones but not in astrocytes in primary culture. J Neurochem 81:207–217. 12064468 10.1046/j.1471-4159.2002.00827.x

[glia22900-bib-0002] Amaral AI , Alves PM , Teixeira AP. 2014 Metabolic fux analysis tools to investigate brain metabolism in vitro In: Hirrlinger J, Waagepertersen HS, editor. Brain energy metabolism. New York: Humana Press pp 107–144.

[glia22900-bib-0003] Amaral AI , Meisingset TW , Kotter MR , Sonnewald U. 2013 Metabolic aspects of neuron‐oligodendrocyte‐astrocyte interactions. Front Endocrinol (Lausanne) 4:54 23717302 10.3389/fendo.2013.00054 PMC3651962

[glia22900-bib-0004] Amaral AI , Teixeira AP , Haakonsen BI , Sonnewald U , Alves AM. 2011a A comprehensive metabolic profile of cultured astrocytes using isotopic transient metabolic flux analysis and ^13^C‐labeled glucose. Front Neuroenerg 3:5 10.3389/fnene.2011.00005 PMC3171112 21941478

[glia22900-bib-0005] Amaral AI , Teixeira AP , Martens S , Bernal V , Sousa MFQ , Alves PM. 2010 Metabolic alterations induced by ischemia in primary cultures of astrocytes: Merging ^13^C NMR spectroscopy and metabolic flux analysis. J Neurochem 113:735–748. 20141568 10.1111/j.1471-4159.2010.06636.x

[glia22900-bib-0006] Amaral AI , Teixeira AP , Sonnewald U , Alves PM. 2011b Estimation of intracellular fluxes in cerebellar neurons after hypoglycemia: Importance of the pyruvate recycling pathway and glutamine oxidation. J Neurosci Res 89:700–710. 21337365 10.1002/jnr.22571

[glia22900-bib-0007] Ariyannur PS , Moffett JR , Madhavarao CN , Arun P , Vishnu N , Jacobowitz DM , Hallows WC , Denu JM , Namboodiri AM. 2010 Nuclear‐cytoplasmic localization of acetyl coenzyme a synthetase‐1 in the rat brain. J Comp Neurol 518:2952–2977. 20533355 10.1002/cne.22373 PMC3047483

[glia22900-bib-0008] Armstrong RC , Mierzwa AJ , Sullivan GM , Sanchez MA. 2015 Myelin and oligodendrocyte lineage cells in white matter pathology and plasticity after traumatic brain injury. Neuropharmacology. pii: S0028‐3908(15)00158‐6 (Epub ahead of print). 10.1016/j.neuropharm.2015.04.029 25963414

[glia22900-bib-0009] Attwell D , Laughlin SB. 2001 An energy budget for signaling in the grey matter of the brain. J Cereb Blood Flow Metab 21:1133–1145. 11598490 10.1097/00004647-200110000-00001

[glia22900-bib-0010] Baer AS , Syed YA , Kang SU , Mitteregger D , Vig R , Ffrench‐Constant C , Franklin RJ , Altmann F , Lubec G , Kotter MR. 2009 Myelin‐mediated inhibition of oligodendrocyte precursor differentiation can be overcome by pharmacological modulation of Fyn‐RhoA and protein kinase C signalling. Brain 132:465–481. 19208690 10.1093/brain/awn334 PMC2640211

[glia22900-bib-0011] Bak LK , Waagepetersen HS , Melo TM , Schousboe A , Sonnewald U. 2007 Complex glutamate labeling from [U‐^13^C]glucose or [U‐^13^C]lactate in co‐cultures of cerebellar neurons and astrocytes. Neurochem Res 32:671–680. 17021949 10.1007/s11064-006-9161-4

[glia22900-bib-0012] Bakken IJ , White LR , Aasly J , Unsgard G , Sonnewald U. 1997 Lactate formation from [U‐^13^C]aspartate in cultured astrocytes: Compartmentation of pyruvate metabolism. Neurosci Lett 237:117–120. 9453229 10.1016/s0304-3940(97)00834-3

[glia22900-bib-0013] Banki K , Colombo E , Sia F , Halladay D , Mattson DH , Tatum AH , Massa PT , Phillips PE , Perl A. 1994 Oligodendrocyte‐specific expression and autoantigenicity of transaldolase in multiple sclerosis. J Exp Med 180:1649–1663. 7964452 10.1084/jem.180.5.1649 PMC2191732

[glia22900-bib-0014] Baslow MH , Guilfoyle DN. 2006 Functions of N‐acetylaspartate and N‐acetylaspartyl‐glutamate in brain—Evidence of a role in maintenance of higher brain integrative activities of information processing and cognition In: Moffett JR, Tieman SB, Weinberger DR, Coyle JT, Namboodiri AMA, editors. N‐acetylaspartate: A unique neuronal molecule in the central nervous system. Bethesda, MD: Springer pp 95–112.

[glia22900-bib-0015] Biemann K. 1962 The mass spectra of isotopically labeled molecules. Mass spectrometry; Organic chemical applications. New York: McGraw‐Hill pp 223–227.

[glia22900-bib-0016] Bolanos JP , Almeida A. 2010 The pentose‐phosphate pathway in neuronal survival against nitrosative stress. IUBMB Life 62:14–18. 19937972 10.1002/iub.280

[glia22900-bib-0017] Brekke EM , Morken TS , Wideroe M , Haberg AK , Brubakk AM , Sonnewald U. 2014 The pentose phosphate pathway and pyruvate carboxylation after neonatal hypoxic‐ischemic brain injury. J Cereb Blood Flow Metab 34:724–734. 24496178 10.1038/jcbfm.2014.8 PMC3982102

[glia22900-bib-0018] Brekke EM , Walls AB , Schousboe A , Waagepetersen HS , Sonnewald U. 2012 Quantitative importance of the pentose phosphate pathway determined by incorporation of ^13^C from [2‐13C]‐ and [3‐13C]glucose into TCA cycle intermediates and neurotransmitter amino acids in functionally intact neurons. J Cereb Blood Flow Metab 32:1788–1799. 22714050 10.1038/jcbfm.2012.85 PMC3434630

[glia22900-bib-0019] Cerdan S , Kunnecke B , Seelig J. 1990 Cerebral metabolism of [1,2‐^13^C_2_]acetate as detected by in vivo and in vitro ^13^C NMR. J Biol Chem 265:12916–12926. 1973931

[glia22900-bib-0020] Cesar M , Hamprecht B. 1995 Immunocytochemical examination of neural rat and mouse primary cultures using monoclonal antibodies raised against pyruvate carboxylase. J Neurochem 64:2312–2318. 7722517 10.1046/j.1471-4159.1995.64052312.x

[glia22900-bib-0021] Chakraborty G , Mekala P , Yahya D , Wu G , Ledeen RW. 2001 Intraneuronal N‐acetylaspartate supplies acetyl groups for myelin lipid synthesis: Evidence for myelin‐associated aspartoacylase. J Neurochem 78:736–745. 11520894 10.1046/j.1471-4159.2001.00456.x

[glia22900-bib-0022] Cruz F , Scott SR , Barroso I , Santisteban P , Cerdan S. 1998 Ontogeny and cellular localization of the pyruvate recycling system in rat brain. J Neurochem 70:2613–2619. 9603228 10.1046/j.1471-4159.1998.70062613.x

[glia22900-bib-0023] Dugas JC , Tai YC , Speed TP , Ngai J , Barres BA. 2006 Functional genomic analysis of oligodendrocyte differentiation. J Neurosci 26:10967–10983. 17065439 10.1523/JNEUROSCI.2572-06.2006 PMC6674672

[glia22900-bib-0024] Dusick JR , Glenn TC , Lee WN , Vespa PM , Kelly DF , Lee SM , Hovda DA , Martin NA. 2007 Increased pentose phosphate pathway flux after clinical traumatic brain injury: A [1,2‐^13^C_2_]glucose labeling study in humans. J Cereb Blood Flow Metab 27:1593–1602. 17293841 10.1038/sj.jcbfm.9600458

[glia22900-bib-0025] Edmond J , Robbins RA , Bergstrom JD , Cole RA , de Vellis J. 1987 Capacity for substrate utilization in oxidative metabolism by neurons, astrocytes, and oligodendrocytes from developing brain in primary culture. J Neurosci Res 18:551–561. 3481403 10.1002/jnr.490180407

[glia22900-bib-0026] Francis JS , Strande L , Markov V , Leone P. 2012 Aspartoacylase supports oxidative energy metabolism during myelination. J Cereb Blood Flow Metab 32:1725–1736. 22617649 10.1038/jcbfm.2012.66 PMC3434629

[glia22900-bib-0027] Funfschilling U , Supplie LM , Mahad D , Boretius S , Saab AS , Edgar J , Brinkmann BG , Kassmann CM , Tzvetanova ID , Mobius W , Diaz F, Meijer D, Suter U, Hamprecht B, Sereda MW, Moraes CT, Frahm J, Goebbels S, Nave KA. 2012 Glycolytic oligodendrocytes maintain myelin and long‐term axonal integrity. Nature 485:517–521. 22622581 10.1038/nature11007 PMC3613737

[glia22900-bib-0028] Gamberino WC , Berkich DA , Lynch CJ , Xu B , LaNoue KF. 1997 Role of pyruvate carboxylase in facilitation of synthesis of glutamate and glutamine in cultured astrocytes. J Neurochem 69:2312–2325. 9375662 10.1046/j.1471-4159.1997.69062312.x

[glia22900-bib-0029] Garcia‐Nogales P , Almeida A , Bolanos JP. 2003 Peroxynitrite protects neurons against nitric oxide‐mediated apoptosis. A key role for glucose‐6‐phosphate dehydrogenase activity in neuroprotection. J Biol Chem 278:864–874. 12414804 10.1074/jbc.M206835200

[glia22900-bib-0030] Geddes JW , Wood JD. 1984 Changes in the amino acid content of nerve endings (synaptosomes) induced by drugs that alter the metabolism of glutamate and gamma‐aminobutyric acid. J Neurochem 42:16–24. 6139419 10.1111/j.1471-4159.1984.tb09691.x

[glia22900-bib-0031] Gegelashvili G , Schousboe A. 1997 High affinity glutamate transporters: Regulation of expression and activity. Mol Pharmacol 52:6–15. 9224806 10.1124/mol.52.1.6

[glia22900-bib-0032] Gegelashvili G , Schousboe A. 1998 Cellular distribution and kinetic properties of high‐affinity glutamate transporters. Brain Res Bull 45:233–238. 9510415 10.1016/s0361-9230(97)00417-6

[glia22900-bib-0033] Haberg A , Qu H , Bakken IJ , Sande LM , White LR , Haraldseth O , Unsgard G , Aasly J , Sonnewald U. 1998 In vitro and ex vivo ^13^C‐NMR spectroscopy studies of pyruvate recycling in brain. Dev Neurosci 20:389–398. 9778576 10.1159/000017335

[glia22900-bib-0034] Hassel B , Sonnewald U , Fonnum F. 1995 Glial‐neuronal interactions as studied by cerebral metabolism of [2‐^13^C]acetate and [1‐^13^C]glucose: An ex vivo ^13^C NMR spectroscopic study. J Neurochem 64:2773–2782. 7760058 10.1046/j.1471-4159.1995.64062773.x

[glia22900-bib-0035] Hertz L , Yu A , Svenneby G , Kvamme E , Fosmark H , Schousboe A. 1980 Absence of preferential glutamine uptake into neurons—An indication of a net transfer of TCA constituents from nerve endings to astrocytes? Neurosci Lett 16:103–109. 7052419 10.1016/0304-3940(80)90109-3

[glia22900-bib-0036] Hofmann U , Maier K , Niebel A , Vacun G , Reuss M , Mauch K. 2008 Identification of metabolic fluxes in hepatic cells from transient 13C‐labeling experiments: Part I. Experimental observations. Biotechnol Bioeng 100:344–354. 18095337 10.1002/bit.21747

[glia22900-bib-0037] Kostic M , Zivkovic N , Stojanovic I. 2013 Multiple sclerosis and glutamate excitotoxicity. Rev Neurosci 24:71–88. 23152401 10.1515/revneuro-2012-0062

[glia22900-bib-0038] Kunnecke B , Cerdan S , Seelig J. 1993 Cerebral metabolism of [1,2‐^13^C_2_]glucose and [U‐^13^C_4_]3‐hydroxybutyrate in rat brain as detected by ^13^C NMR spectroscopy. NMR Biomed 6:264–277. 8105858 10.1002/nbm.1940060406

[glia22900-bib-0039] Lee Y , Morrison BM , Li Y , Lengacher S , Farah MH , Hoffman PN , Liu Y , Tsingalia A , Jin L , Zhang PW , Pellerin L, Magistretti PJ, Rothstein JD. 2012 Oligodendroglia metabolically support axons and contribute to neurodegeneration. Nature 26:443–448. 22801498 10.1038/nature11314 PMC3408792

[glia22900-bib-0040] Lyons SA , Kettenmann H. 1998 Oligodendrocytes and microglia are selectively vulnerable to combined hypoxia and hypoglycemia injury in vitro. J Cereb Blood Flow Metab 18:521–530. 9591844 10.1097/00004647-199805000-00007

[glia22900-bib-0041] Martinez‐Hernandez A , Bell KP , Norenberg MD. 1977 Glutamine synthetase: Glial localization in brain. Science 195:1356–1358. 14400 10.1126/science.14400

[glia22900-bib-0042] Mawhinney TP , Robinett RS , Atalay A , Madson MA. 1986 Analysis of amino acids as their tert.‐butyldimethylsilyl derivatives by gas‐liquid chromatography and mass spectrometry. J Chromatogr 358:231–242. 3722299 10.1016/s0021-9673(01)90333-4

[glia22900-bib-0043] McCarthy KD , de Vellis J. 1980 Preparation of separate astroglial and oligodendroglial cell cultures from rat cerebral tissue. J Cell Biol 85:890–902. 6248568 10.1083/jcb.85.3.890 PMC2111442

[glia22900-bib-0044] McKenna M , Gruetter R , Sonnewald U , Waagepetersen HS , Schousboe A. S. 2012 Energy metabolism of the brain In: Brady ST, Siegel GJ, Albers RW, Price DL, editors. Basic neurochemistry: Principles of molecular, cellular, and medical neurobiology, 8th ed., Oxford, UK: Elsevier Academic Press pp 200–231.

[glia22900-bib-0045] McKenna MC , Stevenson JH , Huang X , Tildon JT , Zielke CL , Hopkins IB. 2000 Mitochondrial malic enzyme activity is much higher in mitochondria from cortical synaptic terminals compared with mitochondria from primary cultures of cortical neurons or cerebellar granule cells. Neurochem Int 36:451–459. 10733013 10.1016/s0197-0186(99)00148-5

[glia22900-bib-0046] McKenna MC , Tildon JT , Stevenson JH , Huang X , Kingwell KG. 1995 Regulation of mitochondrial and cytosolic malic enzymes from cultured rat brain astrocytes. Neurochem Res 20:1491–1501. 8789613 10.1007/BF00970599

[glia22900-bib-0047] Melo TM , Nehlig A , Sonnewald U. 2005 Metabolism is normal in astrocytes in chronically epileptic rats: A ^13^C NMR study of neuronal‐glial interactions in a model of temporal lobe epilepsy. J Cereb Blood Flow Metab 25:1254–1264. 15902201 10.1038/sj.jcbfm.9600128

[glia22900-bib-0048] Mifsud G , Zammit C , Muscat R , Di Giovanni G , Valentino M. 2014 Oligodendrocyte pathophysiology and treatment strategies in cerebral ischemia. CNS Neurosci Ther 20:603–612. 24703424 10.1111/cns.12263 PMC6493108

[glia22900-bib-0049] Moffett JR , Arun P , Ariyannur PS , Garbern JY , Jacobowitz DM , Namboodiri AM. 2011 Extensive aspartoacylase expression in the rat central nervous system. Glia 59:1414–1434. 21598311 10.1002/glia.21186 PMC3143213

[glia22900-bib-0050] Moffett JR , Ross B , Arun P , Madhavarao CN , Namboodiri AM. 2007 N‐acetylaspartate in the CNS: From neurodiagnostics to neurobiology. Prog Neurobiol 81:89–131. 17275978 10.1016/j.pneurobio.2006.12.003 PMC1919520

[glia22900-bib-0051] Morken TS , Brekke E , Haberg A , Wideroe M , Brubakk AM , Sonnewald U. 2014 Altered astrocyte‐neuronal interactions after hypoxia‐ischemia in the neonatal brain in female and male rats. Stroke 45:2777–2785. 25052323 10.1161/STROKEAHA.114.005341

[glia22900-bib-0052] Muir D , Berl S , Clarke DD. 1986 Acetate and fluoroacetate as possible markers for glial metabolism in vivo. Brain Res 380:336–340. 3756485 10.1016/0006-8993(86)90231-3

[glia22900-bib-0053] Murin R , Cesar M , Kowtharapu BS , Verleysdonk S , Hamprecht B. 2009 Expression of pyruvate carboxylase in cultured oligodendroglial, microglial and ependymal cells. Neurochem Res 34:480–489. 18686030 10.1007/s11064-008-9806-6

[glia22900-bib-0054] Nilsen LH , Witter MP , Sonnewald U. 2014 Neuronal and astrocytic metabolism in a transgenic rat model of Alzheimer's disease. J Cereb Blood Flow Metab 34:906–914. 24594625 10.1038/jcbfm.2014.37 PMC4013773

[glia22900-bib-0055] Norenberg MD , Martinez‐Hernandez A. 1979 Fine structural localization of glutamine synthetase in astrocytes of rat brain. Brain Res 161:303–310. 31966 10.1016/0006-8993(79)90071-4

[glia22900-bib-0056] Olstad E , Olsen GM , Qu H , Sonnewald U. 2007 Pyruvate recycling in cultured neurons from cerebellum. J Neurosci Res 85:3318−3325. 17304574 10.1002/jnr.21208

[glia22900-bib-0057] Patel MS. 1974 The relative significance of CO_2_‐fixing enzymes in the metabolism of rat brain. J Neurochem 22:717–724. 4152139 10.1111/j.1471-4159.1974.tb04285.x

[glia22900-bib-0058] Pitt D , Werner P , Raine CS. 2000 Glutamate excitotoxicity in a model of multiple sclerosis. Nat Med 6:67–70. 10613826 10.1038/71555

[glia22900-bib-0059] Reubi JC , van den Berg C , Cuenod M. 1978 Glutamine as precursor for the GABA and glutamate trasmitter pools. Neurosci Lett 10:171–174. 19605275 10.1016/0304-3940(78)90030-7

[glia22900-bib-0060] Rinholm JE , Bergersen LH. 2014 White matter lactate–does it matter? Neuroscience 276:109–116. 24125892 10.1016/j.neuroscience.2013.10.002

[glia22900-bib-0061] Sa Santos S , Sonnewald U , Carrondo MJ , Alves PM. 2011 The role of glia in neuronal recovery following anoxia: In vitro evidence of neuronal adaptation. Neurochem Int 58:665–675. 21316414 10.1016/j.neuint.2011.02.005

[glia22900-bib-0062] Sanchez‐Abarca LI , Tabernero A , Medina JM. 2001 Oligodendrocytes use lactate as a source of energy and as a precursor of lipids. Glia 36:321–329. 11746769 10.1002/glia.1119

[glia22900-bib-0063] Schoenfeld R , Wong A , Silva J , Li M , Itoh A , Horiuchi M , Itoh T , Pleasure D , Cortopassi G. 2010 Oligodendroglial differentiation induces mitochondrial genes and inhibition of mitochondrial function represses oligodendroglial differentiation. Mitochondrion 10:143–150. 20005986 10.1016/j.mito.2009.12.141 PMC3119038

[glia22900-bib-0064] Shank RP , Bennett GS , Freytag SO , Campbell GL. 1985 Pyruvate carboxylase: An astrocyte‐specific enzyme implicated in the replenishment of amino acid neurotransmitter pools. Brain Res 329:364–367. 3884090 10.1016/0006-8993(85)90552-9

[glia22900-bib-0065] Simonishvili S , Jain MR , Li H , Levison SW , Wood TL. 2013 Identification of Bax‐interacting proteins in oligodendrocyte progenitors during glutamate excitotoxicity and perinatal hypoxia‐ischemia. ASN Neuro 5(5):e00131 24195677 10.1042/AN20130027 PMC3891358

[glia22900-bib-0066] Sonnewald U. 2014 Glutamate synthesis has to be matched by its degradation—Where do all the carbons go? J Neurochem 131:399–406. 24989463 10.1111/jnc.12812

[glia22900-bib-0067] Sonnewald U , Rae C. 2010 Pyruvate carboxylation in different model systems studied by ^13^C MRS. Neurochem Res 35:1916–1921. 20842423 10.1007/s11064-010-0257-5 PMC3002159

[glia22900-bib-0068] Sonnewald U , Westergaard N , Hassel B , Muller TB , Unsgard G , Fonnum F , Hertz L , Schousboe A , Petersen SB. 1993a NMR spectroscopic studies of ^13^C acetate and ^13^C glucose metabolism in neocortical astrocytes: Evidence for mitochondrial heterogeneity. Dev Neurosci 15:351–358. 7805589 10.1159/000111355

[glia22900-bib-0069] Sonnewald U , Westergaard N , Schousboe A , Svendsen JS , Unsgard G , Petersen SB. 1993b Direct demonstration by ^13^C NMR spectroscopy that glutamine from astrocytes is a precursor for GABA synthesis in neurons. Neurochem Int 22:19–29. 8095170 10.1016/0197-0186(93)90064-c

[glia22900-bib-0070] Sykes JE , Lopes‐Cardozo M , Van Den Bergh SG. 1986 Relationship between the pentose‐phosphate pathway and the de novo synthesis of fatty acids and cholesterol in oligodendrocyte‐enriched glial cultures. Neurochem Int 8:77–82. 20493032 10.1016/0197-0186(86)90103-8

[glia22900-bib-0071] van den Berg CJ , Garfinkel D. 1971 A stimulation study of brain compartments. Metabolism of glutamate and related substances in mouse brain. Biochem J 123:211–218. 5164952 10.1042/bj1230211 PMC1176925

[glia22900-bib-0072] Waagepetersen HS , Sonnewald U , Larsson OM , Schousboe A. 2001 Multiple compartments with different metabolic characteristics are involved in biosynthesis of intracellular and released glutamine and citrate in astrocytes. Glia 35:246–252. 11494415 10.1002/glia.1089

[glia22900-bib-0073] Yan H , Rivkees SA. 2006 Hypoglycemia influences oligodendrocyte development and myelin formation. Neuroreport 17:55–59. 16361950 10.1097/01.wnr.0000192733.00535.b6

[glia22900-bib-0074] Ziabreva I , Campbell G , Rist J , Zambonin J , Rorbach J , Wydro MM , Lassmann H , Franklin RJ , Mahad D. 2010 Injury and differentiation following inhibition of mitochondrial respiratory chain complex IV in rat oligodendrocytes. Glia 58:1827–1837. 20665559 10.1002/glia.21052 PMC3580049

