# Supplementary figures and images for: Characterization of glucose‐related metabolic pathways in differentiated rat oligodendrocyte lineage cells
Source: Glia. 2015 Sep 9;64(1):21–34. doi: 10.1002/glia.22900 (PMC4832329; doi:10.1002/glia.22900)

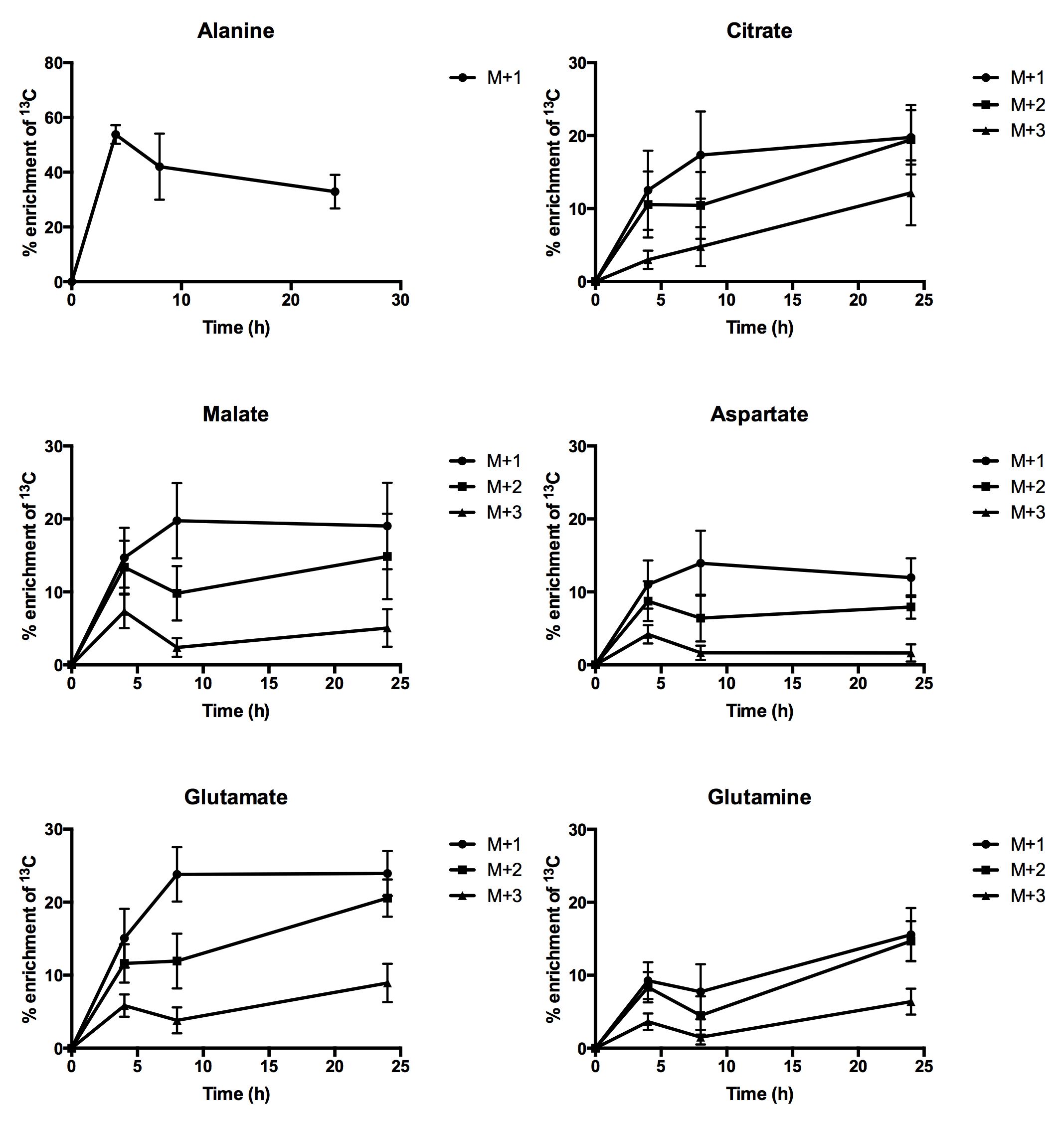

Supplement: Supplementary file 1 — Supporting Information Figure 1 [file GLIA-64-21-s001.tif]
